# Identification and co-expression analysis of long noncoding RNAs and mRNAs involved in the deposition of intramuscular fat in Aohan fine-wool sheep

**DOI:** 10.1186/s12864-021-07385-9

**Published:** 2021-02-01

**Authors:** Fuhui Han, Jing Li, Ranran Zhao, Lirong Liu, Lanlan Li, Qian Li, Jianning He, Nan Liu

**Affiliations:** 1 College of Animal Science and Technology, Qingdao Agricultural University, Qingdao, 266109 China; 2 Qufu Animal Husbandry and Veterinary Technical Service Center, Qufu, 273100 China; 3 China Animal Health and Epidemiology Center, Qingdao, 266032 China

**Keywords:** Intramuscular fat, Aohan fine-wool sheep, Lipid deposition, Long non-coding RNAs, Co-expression analysis

## Abstract

**Background:**

Intramuscular fat (IMF) content has become one of the most important indicators for measuring meat quality, and levels of IMF are affected by various genes. Long non-coding RNAs (lncRNAs) are widely expressed non-coding RNAs that play an important regulatory role in a variety of biological processes; however, research on the lncRNAs involved in sheep IMF deposition is still in its infancy. Aohan fine-wool sheep (AFWS), one of China’s most important meat-hair, dual-purpose sheep breed, provides a great model for studying the role of lncRNAs in the regulation of IMF deposition. We identified lncRNAs by RNA sequencing in Longissimus thoracis et lumborum (LTL) samples of sheep at two ages: 2 months (Mth-2) and 12 months (Mth-12).

**Results:**

We identified a total of 26,247 genes and 6935 novel lncRNAs in LTL samples of sheep. Among these, 199 mRNAs and 61 lncRNAs were differentially expressed. We then compared the structural characteristics of lncRNAs and mRNAs. We obtained target genes of differentially expressed lncRNAs (DELs) and performed enrichment analyses using Gene Ontology (GO) and the Kyoto Encyclopedia of Genes and Genomes (KEGG). We found that target mRNAs were enriched in metabolic processes and developmental pathways. One pathway was significantly enriched, namely tight junction. Based on the analysis of critical target genes, we obtained seven candidate lncRNAs that potentially regulated lipid deposition and constructed a lncRNA-mRNA co-expression network that included MSTRG.4051.3-*FZD4*, MSTRG.16157.3-*ULK1,* MSTRG.21053.3-*PAQR3*, MSTRG.19941.2-*TPI1,* MSTRG.12864.1-*FHL1*, MSTRG.2469.2*-EXOC6* and MSTRG.21381.1-*NCOA1*. We speculated that these candidate lncRNAs might play a role by regulating the expression of target genes. We randomly selected five mRNAs and five lncRNAs to verify the accuracy of the sequencing data by qRT-PCR.

**Conclusions:**

Our study identified the differentially expressed mRNAs and lncRNAs during intramuscular lipid deposition in Aohan fine-wool sheep. The work may widen the knowledge about the annotation of the sheep genome and provide a working basis for investigating intramuscular fat deposition in sheep.

**Supplementary Information:**

The online version contains supplementary material available at 10.1186/s12864-021-07385-9.

## Background

High-quality lamb meat is becoming increasingly popular as living standards improve and dietary patterns change. Currently, evaluations of the meat quality of livestock have revealed that the content of intramuscular fat (IMF) is lower in carcass fats, yet IMF has a critically important influence on the edibility and flavor of muscle meat [1]. Indeed, the quantity of IMF has become one of the most critical parameters of meat quality indicators, as it is considered to be positively related to meat quality and texture [2, 3]. When a certain amount of fat is deposited between the muscle bundles and muscle fibers, the marbled section of the meat has a high score, and the meat is fresh and juicy, which is often considered ideal [4, 5]. The selective deposition of fat can improve production efficiency and play a key role in improving meat quality. This practice is also a major focus and challenge of modern livestock breeding [6]. Therefore, ensuring the appropriate deposition of IMF in lean meat can enhance the future quality of sheep meat.

Studies have shown that intramuscular lipid deposition is affected by multiple genes and signaling pathways, such as the *FAS*, *FAM134B*, and *HSL* genes and the Wnt and AMPK signaling pathways [7–9]. Recently, long non-coding RNAs (lncRNAs) have received increased attention for their wide-ranging functions. LncRNAs refer to a class of non-coding RNAs longer than 200 nt in length [10]. Most lncRNAs have significant temporal and spatial expression specificity [11, 12] and have low sequence conservation among species [13–15]. LncRNAs can be divided into five types based on their positions relative to neighboring protein-coding genes: intronic lncRNAs, bidirectional lncRNAs, sense lncRNAs, intergenic lncRNAs and antisense lncRNAs [16].

LncRNAs can regulate various life activities of the body, including epigenetic regulation, transcriptional regulation and post-transcriptional regulation [17–19]. The most common regulation methods of lncRNAs include cis-regulation of the transcription of neighboring protein-coding genes and the trans-regulation of non-adjacent genes. In addition, lncRNAs can interact with miRNAs to affect the post-transcriptional translation of related mRNAs [20–22]. Studies have shown that lncRNAs can play direct or indirect roles in the process of lipid accumulation [23]. SRA (steroid receptor RNA activator) is one of the earliest discovered lncRNAs and plays an important role in lipid metabolism. SRA can bind to peroxisome proliferator-activated receptor gamma (*PPAR γ*) and enhance *PPAR γ* activity, thereby promoting the differentiation of pre-adipocytes [24]. A study of the expression levels of lncRNAs in the IMF of Jinhua and Landrace pigs revealed a total of 119 differentially expressed lncRNAs (DELs), six of which were involved in fat deposition and lipid metabolism-related pathways [25]. Furthermore, an analysis of transcriptome data from IMF in Inner Mongolia goats revealed that 1472 lncRNAs were involved in adipocyte growth regulation and morphological changes of adipocytes [26]. Another study has shown that lncRNAs can play a key regulatory role in fat deposition in sheep tails [27]. Overall, these findings demonstrate that lncRNAs can regulate lipid deposition through a variety of regulatory mechanisms. However, few studies have assessed the roles of lncRNAs in intramuscular lipid deposition in sheep.

Aohan fine-wool sheep (AFWS) is an important meat-hair, dual-purpose sheep breed in China that grows rapidly early in development. The elimination of male lambs for fat lamb production can increase both hair and meat gains as well as improve the overall benefits provided by fine wool sheep [28]. Exploring the developmental characteristics of IMF deposition and selecting candidate genes for AFWS provide references for future studies and applications in sheep breeding, improve the quality of mutton and accelerate the breeding process. The goal of our study was to systematically identify the profiles of differentially expressed mRNAs (DEMs) and DELs during intramuscular lipid deposition in sheep through high-throughput sequencing. We hoped that by studying the relationship between lncRNAs and lipid deposition, our findings would shed light on the mechanisms underlying selective muscle lipid deposition in sheep.

## Results

### Determination of IMF content

Results for the IMF content of sheep are shown in Table 1. The IMF content of the Longissimus thoracis et lumborum (LTL) at 2, 4, 6 and 12 months was 2.202 ± 0.006, 4.566 ± 0.178, 10.685 ± 0.690 and 11.163 ± 0.878, respectively. We found that the IMF content of LTL at Mth-4 was significantly higher than that at Mth-2 (*P* < 0.01) and was significantly lower than that at Mth-6 and Mth-12 (*P* < 0.01). The IMF content of LTL in Mth-12 was also significantly higher than that observed in Mth-2 (*P* < 0.01). No significant differences were detected between Mth-6 and Mth-12. The same pattern was observed for the biceps femoris muscle (BFM). IMF content in the LTL was significantly higher than that in the BFM in the same month (*P* < 0.01). Thus, Mth-2 and Mth-12 were selected for RNA sequencing (RNA-seq).

**Table 1:** IMF content of sheep (%)

Age	Longissimus thoracis et lumborum IMF(%)	Biceps femoris IMF(%)
**Mth-2**	2.202 ± 0.006^Aa**^	2.012 ± 0.058^Aa^
**Mth-4**	4.566 ± 0.178^Bb**^	3.390 ± 0.149^Bb^
**Mth-6**	10.685 ± 0.690^Cc**^	7.925 ± 0.378^Cc^
**Mth-12**	11.163 ± 0.878^Cc*^	8.867 ± 0.188^Cc^

**IMF content in different parts of muscles among sheep with the same age.** ** indicates that means were highly significantly different (*P* < 0.01); * indicates significant differences (*P* < 0.05); different lowercase letters indicate that means differ significantly (*P* < 0.05) between the same muscles groups of sheep of different ages; different capital letters indicate that means were highly significantly different (*P* < 0.01) between the same muscles groups of sheep of different ages

### Profiles of lncRNAs and mRNAs in sheep muscle

A total of six RNA expression profiles were generated in this experiment. The results are shown in Table 2. The average raw reading was 13.62 G (1G means 1*10^9^ base). After preprocessing the raw data, the average value of the filtered data obtained in each library was 12.82 G. The data obtained from the six expression profiles were relatively average with Q20 ≥ 99% and G/C contents ranging from 49 to 53%, indicating that the quality of the filtered data was reliable. The comparison rate between the filtered clean reads and the reference genome was greater than 88% in all six samples, indicating that the experiment was free of contamination and that the experimental results were robust.

**Table 2:** Statistical data derived from RNA Sequencing

	Mth-2-1	Mth-2-2	Mth-2-3	Mth-12–1	Mth-12–2	Mth-12–3
**Raw reads**	90,397,074	89,528,496	88,813,860	90,619,672	95,989,952	89,577,658
	(13.59G)	(14.40G)	(13.44G)	(13.56G)	(13.43G)	(13.32G)
**Valid reads**	85,098,620	84,675,502	81,473,556	85,252,798	91,240,846	85,201,776
	(12.79G)	(13.69G)	(12.78G)	(12.76G)	(12.70G)	(12.22G)
**Valid Ratio(%)**	94.14	94.58	91.74	94.08	95.05	95.11
**Q20%**	99.98	99.98	99.97	99.98	99.98	99.98
**Q30%**	98.02	97.98	97.83	98.03	98.15	98.17
**GC content(%)**	49.5	51	53	49	49	49
**Mapped reads**	76,713,303	74,525,753	69,966,338	77,129,925	83,264,100	77,731,307
	(90.15%)	(88.01%)	(85.88%)	(90.47%)	(91.26%)	(91.23%)
**Expressed genes**	24,537	24,229	24,295	24,605	24,563	24,077
**Unique lncRNAs**	6495	6492	6507	6532	6541	6425

**Fig. 1:**
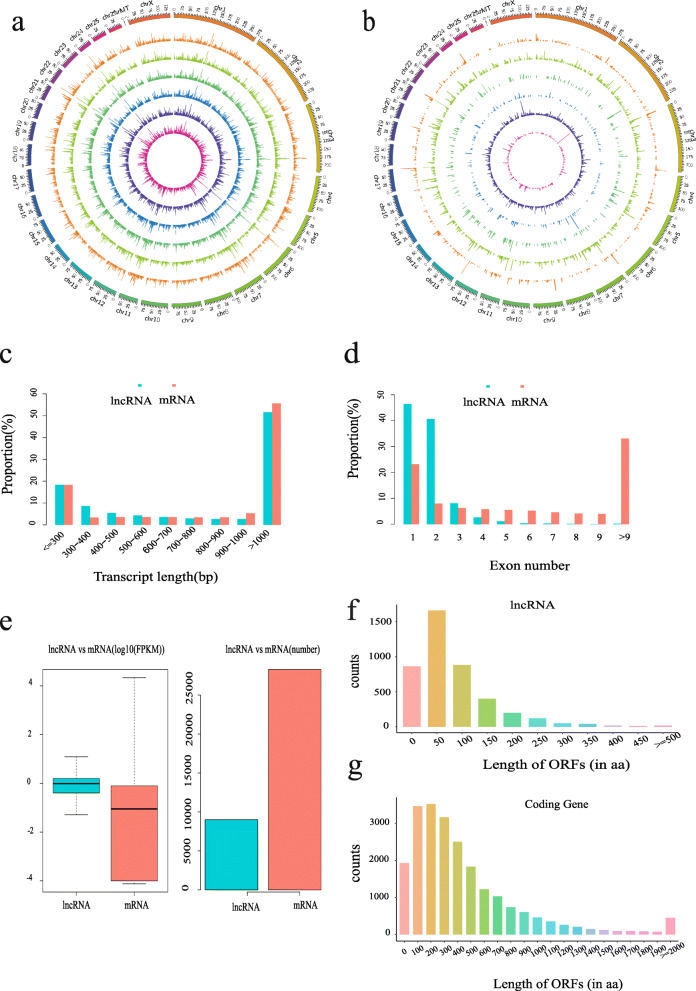
Characteristics of lncRNAs and mRNAs in the intramuscular fat of sheep. **a:** Density distribution of lncRNAs. **b:** Distribution of different types of lncRNAs. **c:** Length distribution of lncRNAs and mRNAs. **d:** Distribution of exon number for lncRNAs and mRNAs. **e:** Expression levels (log_10_FPKM) and numbers of lncRNAs and mRNAs. **f and g:** Length distribution of ORFs of lncRNAs and coding genes

An average of 24,384 expressed genes was identified in the six libraries, and a summary of the protein-coding genes identified is provided in Additional file 1 (Table S1). An average of 6499 unique lncRNAs was identified in the libraries. The information associated with all identified lncRNAs is shown in Additional file 2 (Table S2). We found that the number of reads was positively related to the length of the chromosome (Fig. 1a). Based on the locations of novel lncRNAs in the genome, we identified 525 antisense lncRNAs, 304 sense lncRNAs, 350 bidirectional lncRNAs, 1710 intronic lncRNAs and 4046 intergenic lncRNAs (Fig. 1b). The sequence information for all identified lncRNAs is shown in Additional file 3 (Table S3).

The structural characteristics and the expression levels of lncRNAs and mRNAs were different. The average length of lncRNAs was 868 nt, which was shorter than the average length of mRNAs (2131 nt) (Fig. 1c). LncRNAs consisted of 1.7 exons on average, while mRNAs had 9.9 exons on average (Fig. 1d), thus, lncRNAs had fewer exons than mRNAs. Meanwhile, lncRNAs had lower expression levels relative to mRNAs (Fig. 1e). Moreover, the length of the open reading frame (ORF) of lncRNAs tended to be shorter than that of mRNAs (Fig. 1f, g). Overall, lncRNAs were characterized by shorter lengths, fewer exons, lower expression levels and shorter ORF length distributions compared with mRNAs.

### Identification of differentially expressed mRNAs and lncRNAs

**Fig. 2:**
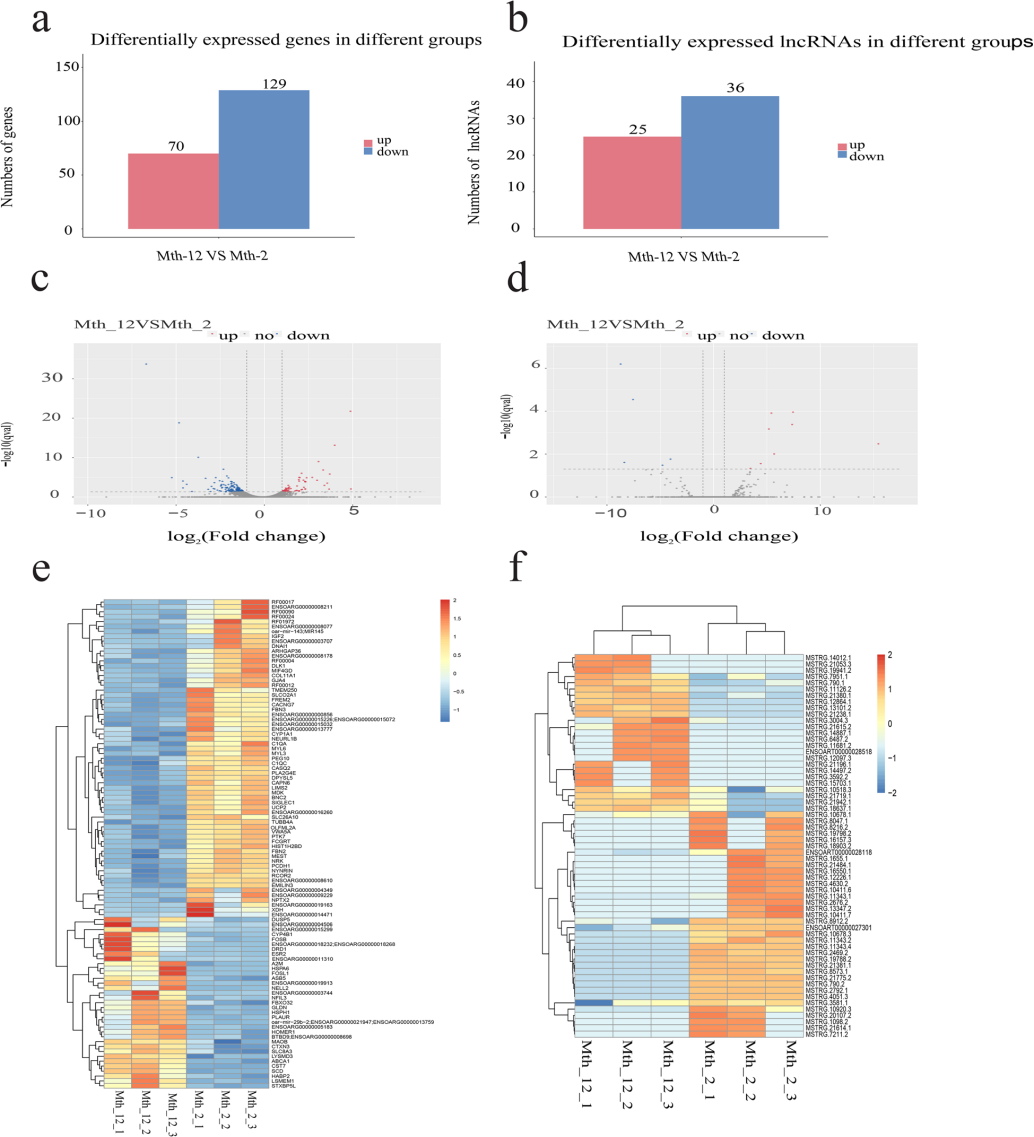
The differentially expressed mRNAs and lncRNAs in the intramuscular fat of sheep. **a** and **b:** The number of up-regulated and down-regulated differentially expressed mRNAs and lncRNAs. The left red bars represent the number of genes up-regulated; the right blue bars represent the number of genes down-regulated. **c** and **d:** The volcano of expressed mRNAs and lncRNAs. The left blue points represent significantly decreased mRNAs and lncRNAs; gray points represent mRNAs and lncRNAs without significant changes. The right red points represent significantly increased mRNAs and lncRNAs. **e and f:** The clustering maps of top 100 differentially expressed mRNAs and lncRNAs. Red indicates that the gene had a higher expression level, and blue indicates that the gene had a lower level of expression

A total of 199 DEMs were identified in muscle tissue (log_2_ (fold change) ≥ 1 or log_2_ (fold change) ≤ − 1 and *q* < 0.05). Of these differentially expressed genes (DEGs), 70 were up-regulated and 129 were down-regulated (Fig. 2a, c). A summary of DEGs is provided in Additional file 4 (Table S4A). We identified 61 lncRNAs that were differentially expressed, of which 25 lncRNAs were up-regulated and 36 lncRNAs were down-regulated (Fig. 2b, d). Interestingly, 58 DELs were novel lncRNAs, we will focus on these novel lncRNAs in subsequent research. The list of DELs is provided in Additional file 4 (Table S4B). To illustrate the distribution of DEGs, we created clustering maps of top 100 DEMs and all the DELs (Fig. 2e, f). Red indicates that the gene had a higher expression level, and blue indicates that the gene had a lower level of expression.

Among the DEMs, we found many genes that have been shown to be related to lipid deposition. *IGF2, CAPN6, UCP2* and *SOCS2* were highly expressed at Mth-2. *IGF2* and *CAPN6* were reported to affect the deposition of intramuscular fat and play an important role in meat efficiency [29, 30]. *UCP2* and *SOCS2* can regulate the proliferation and differentiation of preadipocytes [31, 32]. *FOSB*, *SCD* and *CMYA5* were highly expressed at Mth-12. They have all been found to be potential candidate gene affecting meat quality [33–35]. Among the DELs, we found that MSTRG.13347.2, MSTRG.16157.3, MSTRG.11343.1, MSTRG.11343.4, MSTRG.10678.1 were highly expressed at Mth-2. We speculated that these novel lncRNAs might inhibit lipid deposition. Meanwhile, MSTRG.3004.3, MSTRG.21053.3, MSTRG.14887.1, MSTRG.790.1, MSTRG.10518.3 were highly expressed at Mth-12. We speculated that these novel lncRNAs might promote lipid deposition. However, the regulatory mechanisms underlying these lncRNAs require further study.

### Enrichment analysis of differentially expressed mRNAs

**Fig. 3:**
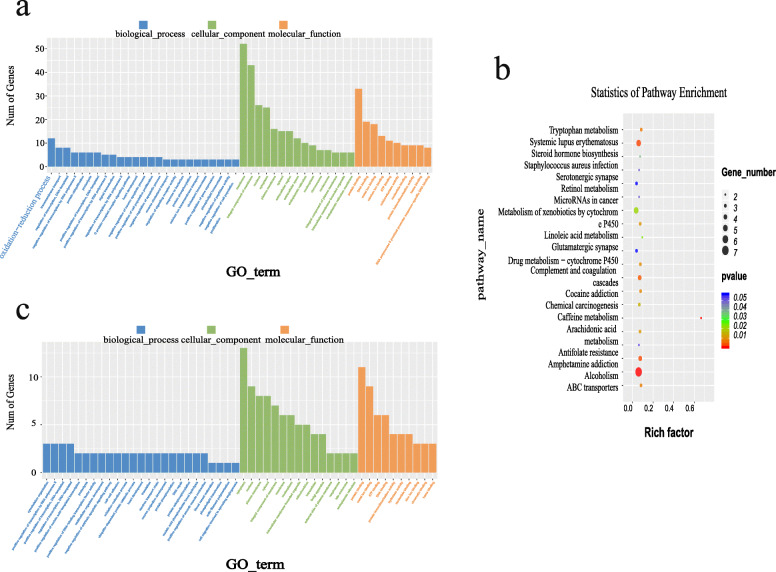
GO terms and pathways analysis of differentially expressed mRNAs and target genes. **a:** The top 25 of biological processes, top 15 of cellular components, top 10 of molecular functions of differentially expressed mRNAs. **b:** Top 20 pathways of differentially expressed mRNAs. The size of the points represents the number of significant differentially expressed mRNAs that matched to a GO term. The color of the points represents the significance of enrichment. **c:** The top 25 of biological processes, top 15 of cellular components, top 10 of molecular functions of differentially expressed target genes

GO functional enrichment analysis of DEGs revealed that these genes participated in a total of 346 significantly enriched functional classifications (*P* < 0.05), 235 of which were related to biological processes, 30 related to cellular components and 81 related to molecular functions (Additional file 5: Table S5A). The top 25 of biological processes, top 15 of cellular components, top 10 of molecular functions were shown in Fig. 3a. The most significantly enriched GO terms were: DNA binding (GO:0003677), extracellular region (GO:0005576), calcium ion binding (GO:0005509), extracellular space (GO:0005615), oxidation-reduction process (GO:0055114), oxidoreductase activity (GO:0016491).

In addition, results of the KEGG pathway analysis showed that these DEGs were involved in 126 biological pathways (Additional file 5: Table S5B), 18 pathways of which were significantly enriched, including arachidonic acid metabolism (ko00590), linoleic acid metabolism (ko00591), steroid hormone biosynthesis (ko00140), and retinol metabolism (ko00830), all of which were related to lipid metabolism. Moreover, the top 20 signaling pathways are shown in Fig. 3b. The results indicated that these pathways may have significantly contributed to the deposition of IMF.

### Comprehensive analysis of candidate lncRNAs and mRNAs

**Fig. 4:**
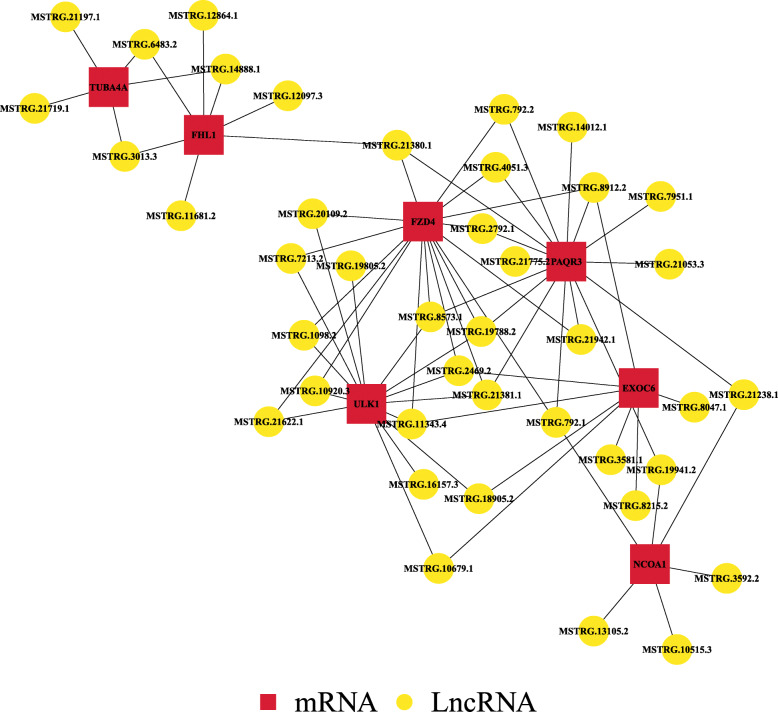
LncRNA-mRNA co-expression network. The red points represent critical mRNAs involved in lipid deposition. The yellow points represent candidate lncRNAs, which can regulate target genes

To understand the potential function of novel lncRNAs, we performed cis-regulation and trans-regulation analyses on candidate lncRNAs. A total of 61 DELs regulated 49 DEMs, all the lncRNAs that acted on 49 mRNAs through trans-regulation (Additional file 6: Table S6). GO analysis of targets of lncRNAs revealed that these genes participated in a total of 422 GO terms, 180 of which were significantly enriched (*P* < 0.05) (Additional file 7: Table S7A). In GO annotation, these DEGs primarily played a role in biological processes. For example, glyceraldehyde-3-phosphate metabolic process (GO:0019682), positive regulation of phospholipid translocation (GO:0061092), glyceraldehyde-3-phosphate biosynthetic process (GO:0046166), cell-cell junction assembly (GO:0007043), muscle cell cellular homeostasis (GO:0046716) and actin filament polymerization (GO:0030041). The top 25 of biological processes, top 15 of cellular components, top 10 of molecular functions were shown in Fig. 3c. The KEGG pathway enrichment analysis of target genes revealed a total of 47 annotated pathways (Additional file 7: Table S7B), of these pathways, one was significantly enriched (*P* < 0.05), namely Tight junction (ko04530). Although some pathways were not significantly enriched, such as Wnt signaling pathway (ko04310), AMPK signaling pathway (ko04152), mTOR signaling pathway (ko04150), Cell adhesion molecules (CAMs)(ko04514) and MAPK signaling pathway (ko04010) and Jak-STAT signaling pathway, these pathways have been reported in the literature to play an important role in lipid deposition [36–41]. Overall, the significantly enriched pathways and GO terms involve 49 target genes. Of these, seven target genes are associated with lipid deposition. There were lncRNAs whose pearson correlation coefficients ≥0.9 or ≤ − 0.9 associated with these seven target genes (Table 3). Of these, seven have high expression levels: MSTRG.16157.3, MSTRG.21053.3, MSTRG.19941.2, MSTRG.2469.2, MSTRG.4051.3, MSTRG.21381.1, MSTRG.12864.1. A network describing the connections between the source genes and lncRNAs (whose pearson correlation coefficients ≥0.8 or ≤ − 0.8) was constructed (Fig. 4). However, the regulatory mechanisms underlying these lncRNAs require further study.

**Table 3:** Critical target mRNAs and candidate lncRNAs related to lipid deposition

Critical target mRNAs	Candidate lncRNAs
**EXOC6**	MSTRG.8047.1/MSTRG.3581.1/MSTRG.8215.2/MSTRG.2469.2
**FZD4**	MSTRG.21381.1/MSTRG.8573.1/MSTRG.4051.3/MSTRG.19788.2/MSTRG.1098.2/MSTRG.21622.1/MSTRG.2792.1/MSTRG.792.2/MSTRG.7213.2/MSTRG.8912.2
**NCOA1**	MSTRG.3592.2/MSTRG.21238.1
**PAQR3**	MSTRG.7951.1/MSTRG.792.1/MSTRG.21053.3/MSTRG.21238.1/MSTRG.2792.1/MSTRG.792.2
**ULK1**	MSTRG.10920.3/MSTRG.7213.2/MSTRG.20109.2/MSTRG.21622.1/MSTRG.1098.2/MSTRG.19788.2/MSTRG.16157.3
**FHL1**	MSTRG.6483.2/MSTRG.14888.1/MSTRG.6483.2/MSTRG.12097.3/MSTRG.12864.1/MSTRG.3013.3
**TPI1**	MSTRG.19941.2/MSTRG.21238.1/MSTRG.7951.1/MSTRG.792.1/MSTRG.21053.3

### Validation of lncRNA and mRNA expression by qRT-PCR

**Fig. 5:**
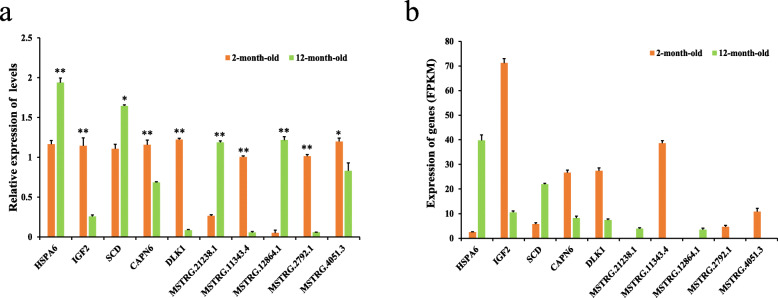
Five differentially expressed mRNAs and five differentially expressed lncRNAs, which were detected by qRT-PCR. **a:** Expression levels of genes by qRT-PCR. **b:** Expression levels of genes by RNA-seq

To validate the expression levels of DELs and DEMs, we randomly selected five DELs and five DEMs and detected their expression levels by qRT-PCR (Fig. 5a). The results of RNA-seq are shown in Fig. 5b. Comparison of the two sets of results above revealed consistent regulatory trends of genes detected by the two methods, indicating that the RNA-seq data were accurate.

## Discussion

IMF content increased gradually with growth, as significant differences were detected between Mth-2 and Mth-12. These findings were consistent with a previous study showing that the IMF content of sheep increased from 0 to 6 months but remained stable thereafter until 12 months of age [42]. Furthermore, these findings are consistent with the characteristics of muscle growth and the development of experimental sheep. The sheep switched to a fattening phase after weaning at 2 months. The weight of sheep increased rapidly between the ages of 4 to 6 months, after which weight gain stabilized. IMF is an important feature contributing to meat quality. Therefore, we selected the LTL samples at Mth-2 (less lipid deposition) and Mth-12 (more lipid deposition) for RNA-seq to provide a robust test of gene expression differences.

Overall, we identified a total of 26,247 genes and 6935 predicted novel lncRNAs in LTL samples of sheep by RNA-seq. Among these, 199 mRNAs (70 up-regulated and 129 down-regulated) and 61 lncRNAs (25 up-regulated and 36 down-regulated) were differentially expressed. We found many DEMs that have been shown to be related to lipid deposition. A marker-derived gene network reveals *CAPN6* can regulate intramuscular fat deposition of beef cattle as modulators of carcass and meat quality traits [30]. Transcriptome analyses examining IMF content in the LTL in heavy Iberian Pigs identified *FOSB* as a candidate gene and other regulatory factors [33]. A study examining gene expression differences in metabolism and function between intramuscular and subcutaneous adipocytes in cattle found that *SCD* was highly expressed in adipocytes and closely associated with fat formation [34]. Similarly, we found many factors that affect the differentiation of pre-adipocytes, such as *UCP2* and *SOCS2*. As the target gene of miR-132–3p, *UCP2* can regulate the differentiation of sheep precursor fat cells [31]. *SOCS2* can also act as a regulator of adipocyte size [32]. However, there are still many DEMs whose functions are unknown, and whether they are related to lipid deposition still needs further research.

To further characterize the mechanisms underlying DEGs, we performed GO and KEGG analysis of DEMs. In GO annotation, these DEGs primarily played a role in biological processes. These processes were closely related to fat formation and deposition, such as reverse cholesterol transport (GO: 0043691), positive regulation of cholesterol efflux (GO:0010875), glyceraldehyde-3-phosphate metabolic process (GO:0019682), positive regulation of phospholipid translocation (GO:0061092), glyceraldehyde-3-phosphate biosynthetic process (GO:0046166) and positive regulation of cholesterol esterification (GO:0010873) [43–48]. In addition, we found that many genes were enriched in biological processes, such as signal transmission, organ development, biosynthesis and cell proliferation, and these are also important processes in muscle development. KEGG pathway analysis revealed that the DEMs were significantly enriched in the immune system, inflammatory response and biological metabolism pathways, demonstrating that signal transmission between adipocytes and immune cells can greatly affect the function of adipose tissue [49]. This result was consistent with the fact that inflammatory cell infiltration has been documented to commonly occur in adipose tissue and stimulate the activation of the immune defense system [50]. We also found that many pathways related to lipid metabolism (Cholesterol metabolism and Arachidonic acid metabolism) were significantly enriched for many DEMs, such as *HABP2*, *CST6, DLK1, PLA2G4E* and *FOSL1* that have been reported to participate in lipid metabolism [51–55]. Based on the GO and KEGG analysis, we obtained DEMs expression profiles that affected the IMF deposition of sheep.

In our study, 61 DELs participated in the regulation of target mRNAs, among them, 58 lncRNAs were novel lncRNAs and all of them acted on 49 target genes through trans-regulation. The functionality of lncRNA is reflected through the study of their target genes [56]. We performed GO and KEGG enrichment analysis on these target genes. We focused on the GO terms related to lipid deposition. These included terms under biological processes, such as glyceraldehyde-3-phosphate metabolic process (GO:0019682), positive regulation of phospholipid translocation (GO:0061092), glyceraldehyde-3-phosphate biosynthetic process (GO:0046166) and cell-cell junction assembly (GO:0007043), as well as molecular functions, such as Wnt-protein binding (GO:0017147), Atg1/ULK1 kinase complex (GO:1990316) and eukaryotic translation initiation factor 2B complex (GO:0005851). We found that the identified lncRNAs might be related to these GO terms. However, further research is required to identify the precise mechanisms.

The KEGG enrichment analysis revealed that target genes were significantly enriched in one pathway, Tight junction (ko04530). Tight junctions have been reported that it has a regulatory effect on the communication of various substances and conditions between cells [57]. Recently, two novel aspects were found that one was their involvement in signal transduction and the other was that tight junctions were considered to be a crucial component of innate immunity [58]. Autophagosome affects metabolism of various substances the by selectively degrading lysosomal targets [59]. Although some signaling pathways were not significantly enriched in our study, such as the “Wnt signaling pathway” “MAPK signaling pathway” and “AMPK signaling pathway”, they are critically important in the process of lipid deposition. Myriad studies have demonstrated the crucial role of canonical Wnt/β-catenin cascade in the development of organs physiological homeostasis and biological metabolism [60]. Wnt/β-catenin signaling pathway is an important developmental pathway that negatively regulates adipogenesis [61]. MAPK/ PI3K/Akt signaling pathway can improve glucose and lipid metabolism via regulation of the metabolic profiling [40]. The AMPK signaling pathway coordinates cell growth, autophagy and metabolism [62]. The above analysis and KEGG pathways might play important roles in lipid deposition and deserve further study.

To facilitate future studies of the mechanisms underlying lncRNAs, we constructed the lncRNA-mRNA co-expression network contained 41 novel lncRNAs and seven mRNAs based on analysis of critical target genes. The seven pairs of lncRNAs-mRNAs included MSTRG.4051.3-*FZD4*, MSTRG.16157.3-*ULK1,* MSTRG.21053.3-*PAQR3*, MSTRG.19941.2-*TPI1,* MSTRG.12864.1-*FHL1*, MSTRG.2469.2*-EXOC6* and MSTRG.21381.1-*NCOA1*. Target genes of these lncRNAs have been reported to be involved in lipid deposition. *FZD4* is highly expressed during fat production [63] and *ULK1* participates in lipid metabolism [64]. *PAQR3* has modulatory roles in obesity, energy metabolism, and leptin signaling [65]. *TPI1* as a glycolytic enzyme, can catalyze the interconversion of dihydroxy-acetone phosphate and glyceraldehyde 3-phosphate in the glycolytic pathway [66]. It was proposed as a potential biomarker for IMF [67]. *FHL1* regulates gene transcription, cell proliferation, metabolism and apoptosis, and plays a role in fat deposition [68]. *Exoc6* participates in GLUT4 translocation in adipocytes thereby affecting glucose transport in fat and muscle cells [69]. Evidence has shown that the p160 co-activator family, consisting of *NCOA1, NCOA2* and *NCOA3*, plays a critical role in adipogenesis and might have a concordant effect on lipid metabolism in mammals [70]. These results provide information for future studies examining how lncRNAs regulate IMF deposition in sheep. The specific regulatory mechanisms require further study and testing.

## Conclusions

Our study systematically identified mRNA and lncRNA expression profiles during intramuscular lipid deposition in sheep. We obtained a total of 199 DEMs and 61 DELs and identified some important lncRNAs related to lipid deposition through GO and KEGG enrichment analysis. In addition, co-expression network analysis of lncRNAs and mRNAs involving 41 lncRNAs and seven mRNAs was conducted based on significant KEGG pathways. Seven pairs of lncRNA-mRNA, including MSTRG.4051.3-*FZD4*, MSTRG.16157.3-*ULK1,* MSTRG.21053.3-*PAQR3*, MSTRG.19941.2-*TPI1,* MSTRG.12864.1-*FHL1*, MSTRG.2469.2*-EXOC6* and MSTRG.21381.1-*NCOA1* were selected for further research. Our study provided a list of the lncRNAs and mRNAs related to intramuscular lipid deposition and laid a foundation for future research on the regulatory mechanisms of lncRNA on sheep muscle lipid deposition.

## Methods

### Sample preparation

All experimental sheep came from the AFWS Stud Farm (Chifeng, Inner Mongolia, China). All sheep were fed under the same feeding and management conditions. A total of 12 healthy AFWS rams (3 individuals for each stage) at 2, 4, 6 and 12 months of age were killed for the sample collection. AFWS rams were obtained from 12 ewes of a similar age and weight that were in estrus simultaneously and were artificially inseminated from the same ram. The 12 healthy AFWS rams were placed in a closed chamber and anesthetized with sodium pentobarbital at a dose of 25 mg/kg by intravenous injection. Rams were anesthetized and eventually sacrificed in the enclosed chamber by having it filled with 20% carbon dioxide every minute until the gas concentration had reached 80%. Experimental animal handling procedures were performed following published protocols [71, 72]. Samples of the LTL and BFM were collected for RNA extraction, placed in RNAase-free Eppendorf tubes and stored immediately in liquid nitrogen. All samples were then stored at − 80 °C until analysis. Likewise, the samples for IMF content determination (150 g per muscle) were packed in plastic bags with ice and stored at − 20 °C in time.

### Determination of IMF content

Soxhlet petroleum-ether extraction was used for the determination of IMF content. After removing the white intermuscular fat from the muscle samples, the samples were minced thoroughly with a meat grinder, loaded into glassware and dried at 105 °C until completely dry. Samples were then weighed after crushing (marked as z), wrapped with quantitative filter paper and baked at 105 °C until samples were dry and their weight did not change. Samples were then weighed in paper bags after drying (marked as x). The dried paper bag was then placed in the Soxhlet extraction bottle, and the ether reflux device was used to reflux the sample at 65 °C until the drops are transparent. The paper bag was then placed in a fume hood to fully volatilize the ether, followed by drying at 105 °C until the weight did not change (marked as y). The measurement was repeated three times for each sample. The following formula was used to calculate the IMF content: IMF content (%) = (x–y) / z × 100%.

### RNA extraction and quality assessment

Total RNA of the LTL was extracted using Trizol reagent (Invitrogen, Carlsbad, CA, USA) according to the manufacturer’s instructions. RNA purity was measured at an OD 260/280 with a NanoDrop ND-2000 instrument (Thermo Fisher Scientific, MA, USA). RNA integrity (RIN) was evaluated by 1% agarose gel electrophoresis and Agilent 2100 bioanalyzer (Agilent, Santa Clara, CA, USA). RNA samples with OD_260_/OD_280_ ratio greater than 1.8 and RIN value greater than 7.5 were selected for sequencing.

### Library preparation and sequencing

First-strand complementary DNA (cDNA) was synthesized using random hexamer primers and M-MuLV reverse transcriptase (RNase H-) [73], with rRNA-depleted RNA used as a template. Second-strand cDNA was then synthesized with dNTPs, DNA polymerase I and RNase H. Next, T4 DNA polymerase and Klenow DNA polymerase were used to repair and modify the ends to add an A base and ligate the sequencing adapter. The cDNA products were then purified using AMPure XP beads (Beckman Coulter, Brea, CA, USA). Finally, uracil DNA glycosylase (NEB, Ipswich, MA, USA) was used to degrade the U-containing chain to remove second-strand cDNA. The purified first-strand cDNA was enriched by PCR to obtain a cDNA library. The quality of the libraries was assessed using an Agilent 2100 Bioanalyzer, and sequencing was performed using paired-end sequencing (2*150 bp) with the Illumina HiSeq 4000 platform (LC Sciences, Houston, TX, USA).

### Mapping of reads and transcriptome assembly

Cutadapt (Version 1.10) was used to remove the reads that were contaminated by adapters, low-quality bases and undetermined bases. The clean reads were mapped to the reference genome *Ovis aries* Ensembl release 96 (ftp://ftp.ensembl.org/pub/release-96/fasta/ovis_aries/dna/) using HISAT2(Version 2.0.4) [74]. Mapped reads of each sample were assembled by StringTie (Version 1.3.0) [75]. Gffcompare (http://ccb.jhu.edu/software/stringtie/gffcompare.shtml) was used to combine all transcripts from samples to reconstruct a comprehensive transcriptome. StringTie was used to determine the expression level for all transcripts by calculating FPKM (FPKM = [total exon fragments/mapped reads (millions) × exon length (kb)]).

### Identification of lncRNAs

Known transcripts and transcripts less than 200 bp in length were removed from the data set. CPC (Coding Potential Calculator, Version 0.9-r2) and CNCI (Coding-Non-Coding Index, Version 2.0) were then used to screen lncRNAs [76, 77]. When the CPC software score was less than 0.5 and the CNCI software score was less than 0, a transcript was considered a novel lncRNA. We used circos (http://www.circos.ca) software to perform genomic mapping on the lncRNAs obtained by screening. The *P*-value was adjusted using the Benjamini & Hochberg method [78]. A corrected *P*-value of 0.05 was set as the threshold for significantly differential expression. The R package “edge R”(Version 3.4.4) was used for difference statistics and visual drawing, which was used to select the differentially expressed transcripts that satisfied the condition of log_2_ (fold change) ≥ 1 or log2 (fold change) ≤ − 1 and *q* < 0.05.

### Enrichment analysis of differentially expressed mRNAs

We used the Gene Ontology database (http://www.geneontology.org) and the Kyoto Encyclopedia of Genes and Genomes (http://www.kegg.jp/kegg) to annotate DEGs. The genes were mapped to GO terms and KEGG pathways based on annotation information and then the hypergeometric test was performed. The clustering map was drawn by the R package “edge R”. GO terms and KEGG pathways were defined as significantly enriched when *P* < 0.05.

### Prediction of lncRNA target genes

Based on the cis-and trans-regulation mechanisms of lncRNA, we identified the protein-coding genes (100-kb upstream and downstream) located on the same chromosome as the lncRNA that was a target for cis-regulation. RIsearch (https://rth.dk/resources/risearch/) was used to predict the free energy of lncRNA-mRNA gene combinations on different chromosomes; combinations of lncRNA and mRNA with free energies below − 11 kcal/mol were identified as trans target genes of lncRNA [79]. The results of the cis and trans-regulation were used to calculate Pearson correlations between lncRNA and mRNA expression. Cytoscape was used to plot the co-expression network.

### Verification of sequencing data

We randomly selected five lncRNAs and five mRNAs to validate their expression using SYBR Green PCR Master Mix (Takara, Dalian, China). Primer 5 (http://www.premierbiosoft.com/index.html) was used to design primers for the candidate genes. The sequences of the primers used are listed in Additional file 8 (Table S8). A 20-μL PCR mixture consisted of 10 μL SYBR® Premix Ex Taq II (2×), 0.5 μL forward primer (10 μM/L), 0.5 μL reverse primer (10 μM/L), 1 μL cDNA and 8 μL ddH_2_O. The PCR parameters were as follows: 95 °C for 30 s; 40 cycles of 95 °C for 5 s; 60 °C for 30 s; 72 °C for 30 s; and 72 °C for 5 min. Three replicates were conducted for each sample. The 2^-ΔΔCt^ method was used to quantify relative expression levels [80].

### Statistical analysis

Data on IMF content were expressed as means± standard deviation. One-way analyses of variance in SPSS 17.0 were used to analyze experimental results. Independent sample t-tests were used to compare the IMF content of muscles at the same age. All the data from the qRT-PCR were obtained using at least three independent replicates. Differences were deemed statistically significant if *P* < 0.05.

## Supplementary Information

**Additional file 1 Table S1.** The summary of protein-coding genes identified in the libraries.

**Additional file 2 Table S2.** The summary of lncRNAs identified in the libraries.

**Additional file 3 Table S3.** Sequence information of all expressed lncRNAs found in the study.

**Additional file 4 Table S4(A).** The summary of differentially expressed protein-coding genes. **Table S4(B).** The summary of differentially expressed lncRNAs.

**Additional file 5 Table S5(A).** GO enrichment analysis of the differentially expressed mRNAs. S gene number: the number of significant differentially expressed mRNAs which match to a GO term; TS gene number: the number of significant differentially expressed mRNAs which have GO annotations; B gene number: the number of detected mRNAs which match to a GO term; TB gene number: the number of all detected mRNAs which have GO annotations. **Table S5(B).** KEGG enrichment analysis (*P* < 0.05) of the differentially expressed mRNAs.

**Additional file 6 Table S6.** The differentially expressed target mRNAs of the differentially expressed lncRNA in trans- regulatory roles.

**Additional file 7 Table S7(A).** GO enrichment analysis of differentially expressed target mRNAs of differentially expressed lncRNAs in the study. **Table S7 (B)**. KEGG enrichment analysis of differentially expressed target mRNAs of differentially expressed lncRNAs in the study.

**Additional file 8 Table S8.** Primers used in the qRT-PCR analysis.

## Data Availability

Additional data can be found in supplementary files. The RNA-Seq data was submitted to the SRA database under accession number SRR12247890.

## Abbreviations

AFWS: Aohan fine wool sheep
IMF: Intramuscular fat
LncRNAs: Long noncoding RNAs
Mth-2, Mth-4, Mth-6, Mth-12: Rams at 2, 4, 6, 12 months
LTL: Longissimus thoracis et lumborum
BFM: Biceps femoris muscle
RNA-seq: RNA sequencing
DEGs: Differentially expressed genes
DEMs: Differentially expressed mRNAs
DELs: Differentially expressed lncRNAs
ORF: Open reading frame
GO: Gene ontology
KEGG: Kyoto encyclopedia of genes and genomes
qRT-PCR: Quantitative real-time PCR
CPC: Coding Potential Calculator
CNCI: Coding-Non-Coding Index
RIN: RNA integrity
